# Roles of monocarboxylate transporter subtypes in promotion and suppression of osteoclast differentiation and survival on bone

**DOI:** 10.1038/s41598-019-52128-2

**Published:** 2019-10-30

**Authors:** Hiroko Imai, Kentaro Yoshimura, Yoichi Miyamoto, Kiyohito Sasa, Marika Sugano, Masahiro Chatani, Masamichi Takami, Matsuo Yamamoto, Ryutaro Kamijo

**Affiliations:** 1 Department of Biochemistry, Showa University School of Dentistry, Tokyo, Japan; 2 Department of Periodontology, Showa University School of Dentistry, Tokyo, Japan; 3 Department of Pharmacology, Showa University School of Dentistry, Tokyo, Japan

**Keywords:** Cell growth, Transporters, RNAi

## Abstract

Monocarboxylate transporters (MCTs) provide transmembrane transport of monocarboxylates such as lactate and pyruvate. The present results showed that α-cyano-4-hydroxycinnamic acid (CHC), an inhibitor of MCTs, promoted osteoclast differentiation from macrophages at lower concentrations (0.1–0.3 mM) and suppressed that at a higher concentration (1.0 mM). On the other hand, CHC reduced the number of mature osteoclasts on the surface of dentin in a concentration-dependent manner. Additionally, macrophages and osteoclasts were found to express the *Mct1*, *Mct2*, and *Mct4* genes, with *Mct1* and *Mct4* expression higher in macrophages, and that of *Mct2* higher in osteoclasts. Although *Mct1* gene knockdown in macrophages enhanced osteoclast formation induced by RANKL, *Mct2* gene knockdown suppressed that. Finally, *Mct2* gene silencing in mature osteoclasts decreased their number and, thereby, bone resorption. These results suggest that MCT1 is a negative regulator and MCT2 a positive regulator of osteoclast differentiation, while MCT2 is required for bone resorption by osteoclasts.

## Introduction

Monocarboxylate transporters (MCTs), a family of proton-linked plasma membrane transporters that carry monocarboxylates across the membrane, have been classified into 14 different subtypes^1^, though only MCT1, MCT2, MCT3, and MCT4 are reported to be involved in the transport of lactate and pyruvate^2^. In mammalian cells, these 4 subtypes have functions related to uptake and efflux of monocarboxylic acids including lactate and pyruvate across the membrane. Lactic acid and ketone bodies are taken up for oxidation into heart and red muscle cells, as well as neurons, through MCT1 which is ubiquitously expressed, or MCT2, expressed in kidney and brain tissues^1,2^. Furthermore, lactic acid is also taken up by cells in the liver and kidneys through MCT1 or MCT2 for gluconeogenesis. On the other hand, efflux of monocarboxylic acid from cells occurs via MCT1, MCT3, and MCT4. Lactic acid is produced by glycolysis under hypoxic conditions in nearly all types of cells, then released to the outside via MCT1 to suppress a decrease in intracellular pH and prevent stoppage of glycolysis by accumulating intracellular lactic acid. MCT4 is also involved in the efflux of lactic acid from cells that produce energy by glycolysis even under normoxic conditions^3,4^. MCT3 has more specific activities, and is expressed only in retinal pigment and choroid plexus epithelium, where it is responsible for transportation of lactic acid to the outside of those cells^1^.

Based on these backgrounds, the MCT1-4 subtypes have been considered to have functions generally related to maintaining the energy metabolism of cells, as well as homeostasis of intracellular pH by the transportation of lactate and other monocarboxylates^5^. However, we recently reported that altering the activity of MCT has other significant effects on cells^6–8^. More specifically, MCT1 was required for cell death induced by IL-1β in mouse chondrocyte-like ATDC5 cells^6^. IL-1β-treated ATDC5 cells expressed 2 different enzymes, namely, inducible NO synthase (iNOS) and phagocyte-type NADPH oxidase (NOX-2). Peroxynitrite, a reaction product of NO and superoxide produced by iNOS and NOX-2, respectively, caused mitochondrial dysfunction, resulting in cell death^7^. We found that *Mct1* silencing reduced intracellular ROS production and late-phase activation of NF-κB, which suppressed NOX-2 expression and cell death^6^.

In another study, we found that silencing of *Mct1* by siRNA strongly suppresses osteoblast differentiation^8^. Using a culture system in which C2C12 cells, a mouse myoblast cell line, were stimulated with BMP-2 to induce differentiation into osteoblasts, *Mct1* silencing suppressed the expression of *Runx2* and *Sp7*, major transcription factors for osteoblast differentiation, as well as that of *Tnap*, a differentiation marker of mature osteoblasts. These changes were caused by increased expression of *Trp53* in C2C12 cells induced by *Mct1* silencing. Together, these findings indicate that MCT1 is involved not only in inflammatory cell death but also normal differentiation of osteoblasts, thus suggesting possible involvement of MCT in bone formation and remodeling.

On the other hand, the roles of MCTs in osteoclasts, the counterpart of osteoblasts for maintaining bone metabolism, have not been elucidated. In the present study, we investigated the roles of MCT subtypes in osteoclast differentiation and function.

## Results

### α-cyano-4-hydroxycinnamic acid (CHC) promoted osteoclast differentiation from BMMs

**Figure 1:**
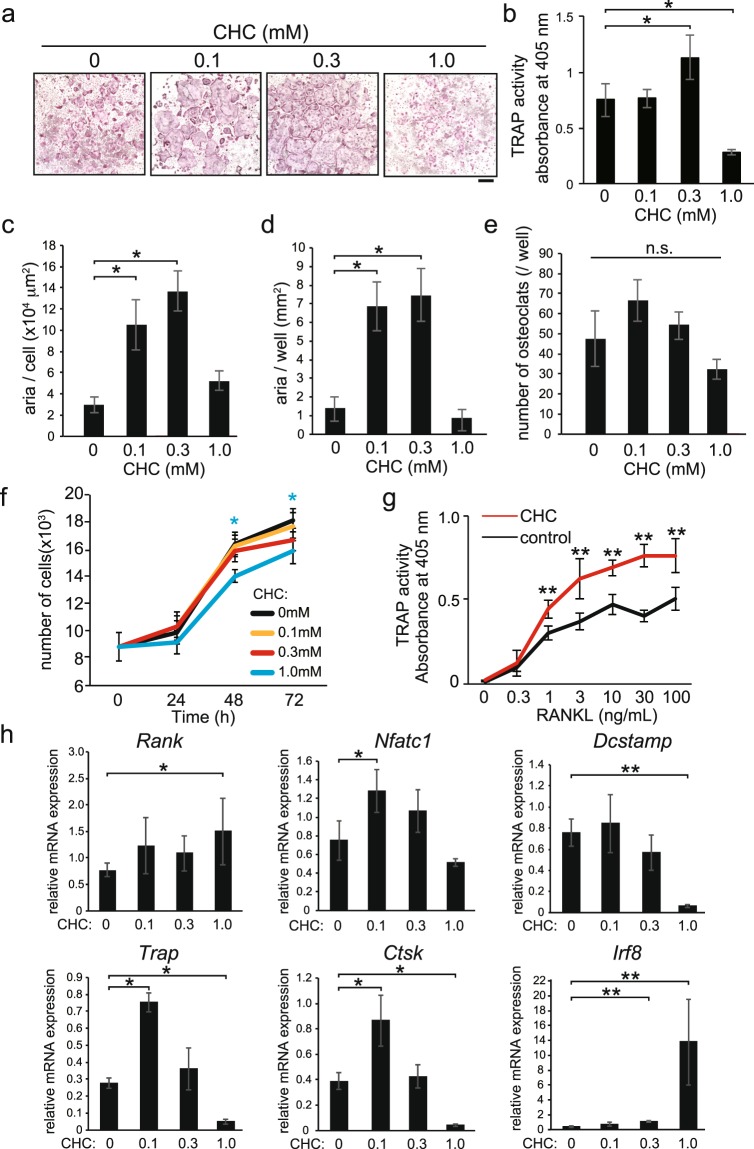
Effect of CHC on RANKL-induced differentiation of BMMs into osteoclasts. (**a–e**) BMMs were cultured for 72 hours in the presence of M-CSF, RANKL, and various concentrations of CHC. (**a**) Osteoclasts were detected by TRAP activity staining. (**b**) TRAP activity in the cultures was determined. (**c**–**e**) The area of osteoclasts (**c**), total osteoclast area (**d**), and the number of osteoclasts (**e**) per well were quantified using the software package pre-installed in the optical microscope. (**f)** BMMs were cultured for 24, 48, and 72 hours in the presence of M-CSF and the indicated concentrations of CHC, which was followed by determination of cell number to evaluate BMM proliferation. (**g**) BMMs were cultured for 72 hours with M-CSF and various concentrations of RANKL in the presence or absence of CHC (0.3 mM). TRAP activity in the cultures was determined. (**h**) BMMs were cultured for 48 hours in the presence of M-CSF, RANKL, and various concentrations of CHC. Expression levels of *Rank*, *Nfatc1*, *Dcstamp*, *Trap*, *Ctsk*, and *Irf8* are expressed as relative to that of *Gapdh*. (**a**) Scale bars, 500 μm. (**b**–**h**) Values are expressed as the mean ± SD. * and ** indicate significant difference at *p* < 0.05 and *p* < 0.01, respectively. n.s., not significant.

Bone marrow macrophages (BMMs), obtained by culturing mouse bone marrow cells (BMCs) in the presence of M-CSF, were further cultured for 72 hours in the presence of M-CSF and RANKL to induce their differentiation into osteoclasts. Addition of CHC known to inhibit MCT1, MCT2, MCT3, and MCT4, promoted RANKL-induced osteoclast formation (Fig. 1a). Additionally, CHC (0.3 mM) increased TRAP activity (Fig. 1b) and osteoclast size (Fig. 1c,d), whereas it had no effect on either number of osteoclasts (Fig. 1e) or proliferation of BMMs (Fig. 1f, red line). Hence, the increased TRAP activity by CHC (0.3 mM) in the presence of various concentrations of RANKL (Fig. 1g) was a result of something other than the number of BMMs. On the other hand, addition of CHC at 1.0 mM inhibited osteoclast formation (Fig. 1a), decreased TRAP activity (Fig. 1b), and suppressed BMM proliferation (Fig. 1f, blue line), whereas it did not have a significant effect on the number of osteoclasts (Fig. 1e).

### CHC changed expression of genes for osteoclast differentiation markers in BMMs

To analyze the effect of CHC on expression of genes for osteoclast differentiation markers induced by RANKL, various concentrations of CHC were added simultaneously with RANKL to the BMM cultures. CHC at 0.1 mM increased the RANKL-induced expression of *Nfatc1, Trap*, and *Ctsk* as compared to the control group (Fig. 1h), which suggested that CHC (0.1 mM) promoted osteoclast differentiation from BMMs. On the other hand, CHC at 1.0 mM decreased the expression levels of *Dcstamp*, *Trap*, and *Ctsk*, while that strongly increased the expression of *Irf8*, a negative regulator of osteoclastgenensis^9^. CHC at 1.0 mM also increased the expression of *Rank* (Fig. 1h).

### CHC reduced the number of mature osteoclasts on dentine

**Figure 2:**
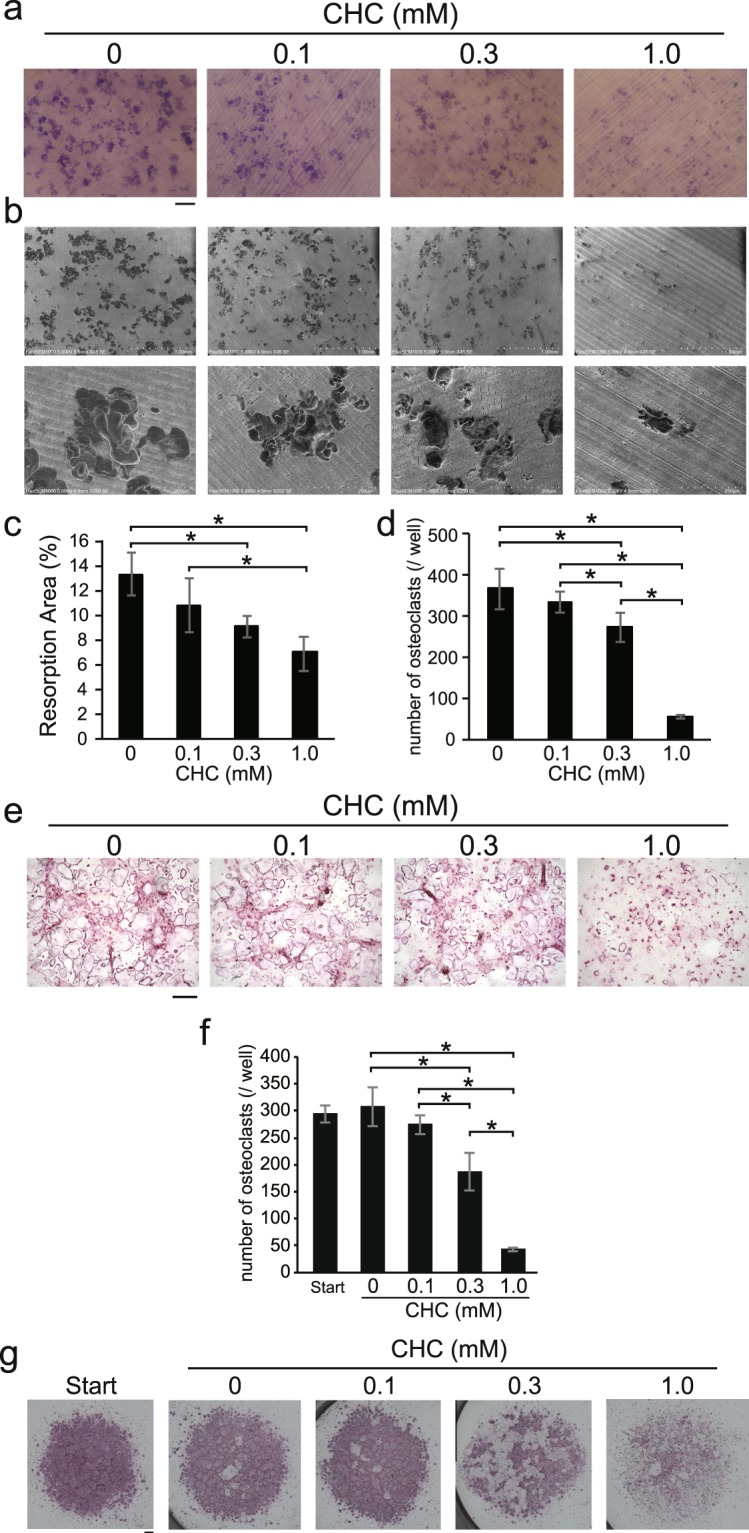
Effect of CHC on the survival of mature osteoclasts attached to the dentin surface. (**a–e**) Osteoblastic UAMS32 cells and BMCs were co-cultured in the presence of 1α,25(OH)_2_D_3_ (10 nM) and PGE_2_ (1 μM) for 8 days in collagen-coated plates. Cells were re-plated on dentin discs (**a**–**c**) or normal plastic plates (**d,e**), then cultured for an additional 24 hours in the presence of various concentrations of CHC. (**a**) Resorption pits formed on dentin discs were stained with toluidine blue. (**b**) The surface of each dentin disc was observed using scanning electron microscopy. Magnification, 45 × (upper panels) and 250 × (lower panels). (**c**) The area of resorption pits stained with toluidine blue was quantified using the software package pre-installed in the optical microscope. (**d**) The number of osteoclasts was determined under a microscope. (**e**) TRAP activity staining of cultures in normal plastic plates was performed. (**f**,**g**) BMMs were cultured for 72 hours in the presence of M-CSF and RANKL to induce differentiated into osteoclasts in normal plastic plates. After counting the initial number of osteoclasts (Start), they were further cultured for 48 hours in the presence or absence of the indicated concentrations of CHC, and the number of osteoclasts remained on the plates was counted (**f**). Osteoclasts were detected by TRAP activity staining (**g**). (**a**,**e**,**g**) Scale bars, 500 μm. (**c**,**d**,**f**) Values are shown as the mean ± SD. *Value significantly smaller than that obtained in the control without CHC (*p* < 0.05).

Since CHC was shown to modulate osteoclast differentiation of BMMs, we next examined the effect of CHC on bone resorption by mature osteoclasts using pit assays. Osteoclasts obtained by co-cultivation of UAMS32 cells with BMCs were seeded on dentin disks and allowed to form resorption pits. Following osteoclast attachment to the disk, CHC was added to the medium, which resulted in reduction of the number of resorption pits in a concentration-dependent manner (Fig. 2a,c). Scanning electron microscopy findings also confirmed that addition of CHC reduced the number of resorption pits (Fig. 2b). In addition, CHC decreased the number of osteoclasts obtained in co-cultures of BMCs and UAMS32 cells in a concentration-dependent manner (Fig. 2d,e). CHC also reduced the number of mature osteoclasts obtained by culturing BMM in the presence of RANKL (Fig. 2f,g). CHC also decreased TRAP activity in osteoclasts that had differentiated from BMMs in calcium phosphate-coated plates in the presence of M-CSF and RANKL (Supplementary Fig. S1a). Furthermore, CHC inhibited the formation of actin rings, required for attachment of osteoclasts to the bone surface for bone resorption (Supplementary Fig. S1b, red arrowhead). On the other hand, CHC did not induce apoptosis in osteoclasts (Supplementary Fig. S1c). These results suggested that CHC reduced the number of mature osteoclasts at least in part through inhibition of the attachment of mature osteoclasts to the plates and the surface of dentin discs.

### BMMs and osteoclasts expressed *Mct1*, *Mct2*, and *Mct4*

**Figure 3:**
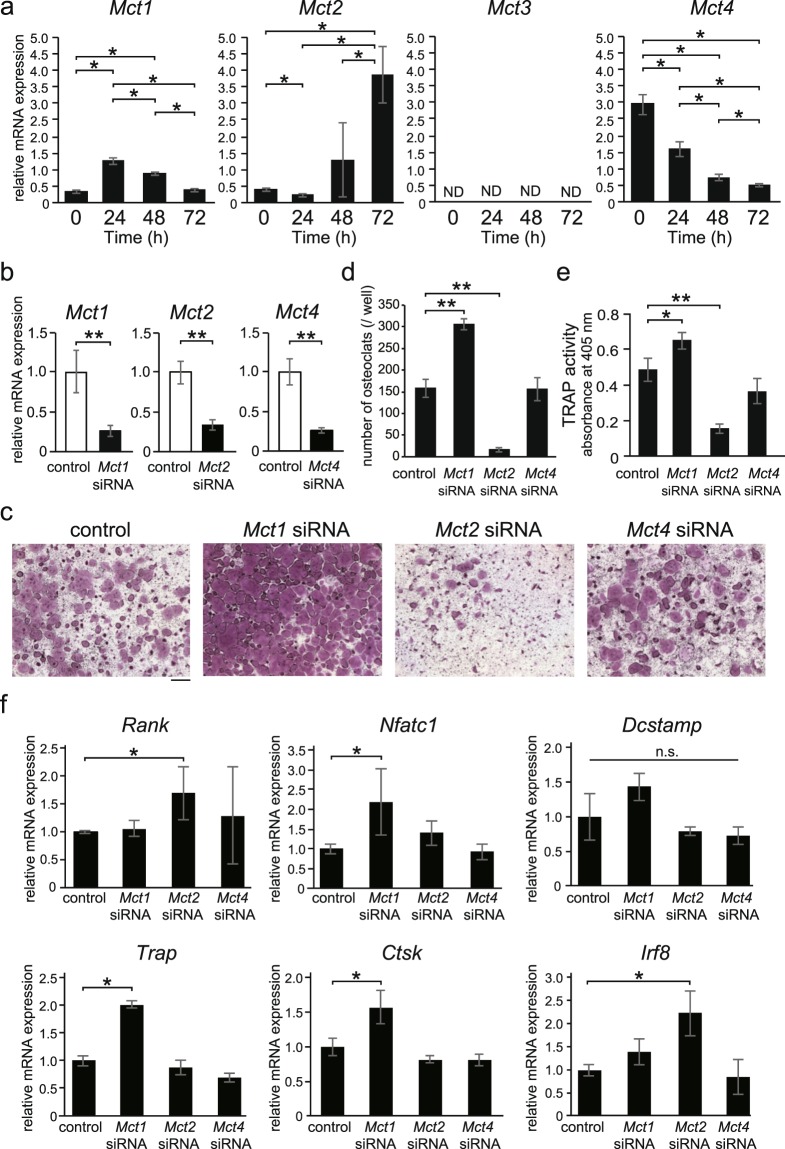
Effects of *Mct1*, *Mct2*, and *Mct4* gene silencing in BMMs on osteoclast differentiation. (**a**) Expression levels of *Mct1*, *Mct2*, *Mct3*, and *Mct4* during the process of RANKL-induced osteoclast differentiation from BMMs. Values are shown as relative to that of *Gapdh*. ND, not detected. **(b**–**f**) *Mct1*, *Mct2*, or *Mct4* siRNA, or control siRNA was introduced into BMMs. (**b**) At 24 hours after introduction of each siRNA, expression of mRNA for *Mct1*, *Mct2*, and *Mct4* was quantitatively analyzed by real-time RT-PCR. Values are normalized to that for *Gapdh* and shown as relative to the control. (**c**–**e**) After introduction of siRNA, cells were cultured for 72 hours in the presence of M-CSF and RANKL. Osteoclasts were detected by TRAP activity staining (**c**) and the number of osteoclasts per well was counted (**d**). TRAP activity in the cultures was determined (**e**). (**f**) BMMs introduced with each siRNA were cultured for 48 hours in the presence of M-CSF and RANKL. The expressions of *Rank*, *Nfatc1*, *Dcstamp*, *Trap*, *Ctsk*, and *Irf8* were normalized to that of *Gapdh*, and expressed as relative values. (**a**,**b**,**d**–**f**) Values are shown as the mean ± SD. * and ** indicate a significant difference at *p* < 0.05 and *p* < 0.01, respectively. (**c**) Scale bar, 500 μm.

In order to clarify the gene expression profile of MCT subtypes in BMMs and osteoclasts, RNA samples were collected every 24 hours after stimulation with RANKL, and real-time PCR analysis was performed for MCT subtypes. *Mct1* expression was increased at 24 hours after stimulation with RANKL and then decreased with the progress of osteoclast differentiation. That of *Mct2* was low in BMMs, then increased with the progression of osteoclast differentiation, while the expression of *Mct4* was high in BMMs and then decreased with differentiation. *Mct3* expression was not detected under the present experimental settings (Fig. 3a). It has been reported that monocarboxylic acid is taken up through MCT1 or MCT2, then flowed out by MCT1, MCT3, or MCT4^1,2^. The present results indicate that BMMs mainly use MCT4 to release monocarboxylic acid from cells, while mature osteoclasts use MCT2 to take up monocarboxylic acid into cells. In addition, it has also been reported that RANKL promotes the glycolytic system in BMMs^10^. Thus, our findings suggest that the transient increase of *Mct1* expression following RANKL stimulation is involved in extracellular release of lactic acid to suppress intracellular acidification and prevent stoppage of glycolysis by accumulation of intracellular lactic acid.

### Osteoclast formation from BMMs promoted by *Mct1* siRNA and suppressed by *Mct2* siRNA

Next, we introduced specific siRNAs for *Mct1*, *Mct2*, and *Mct4* to BMMs to clarify their individual roles in osteoclast differentiation (Fig. 3b). None of the subtype siRNAs had effects on BMM cell proliferation (data not shown). *Mct1* siRNA promoted and *Mct2* siRNA strongly suppressed osteoclast formation, while *Mct4* siRNA had no effect (Fig. 3c,d). Promotion of osteoclast differentiation by *Mct1* siRNA and its suppression by *Mct2* siRNA was also confirmed by determination of TRAP activities (Fig. 3e).

### Introduction of siRNAs for *Mct1* and *Mct2* into BMMs altered expression of osteoclast differentiation marker genes

The effects of introduction of individual siRNAs for *Mct1*, *Mct2*, and *Mct4* into BMMs on the expression of genes related to osteoclast differentiation induced by RANKL (Fig. 3f) were investigated. *Mct1* siRNA increased the expression of *Nfatc1*, a transcription factor essential for osteoclast differentiation^11^, as well as that of *Trap* and *Ctsk*, representative marker genes of osteoclasts^12,13^. These results indicate that MCT1 suppresses osteoclast differentiation through, at least in part, inhibition of *Nfatc1* expression. While *Mct2* siRNA increased the expression of the *Rank* gene, it also augmented the expression of *Irf8*^9^, a transcription factor that negatively regulates osteoclast differentiation, indicating the possibility that MCT2 functions as a promoter of osteoclast differentiation via suppression of *Irf8*. On the other hand, *Mct4* siRNA did not have any effects on the expression of *Rank*, *Nfatc1*, *Dcstamp*, *Trap*, *Ctsk*, or *Ifr8*.

### *Mct2* gene silencing in mature osteoclasts suppressed their survival/attachment to bone

**Figure 4:**
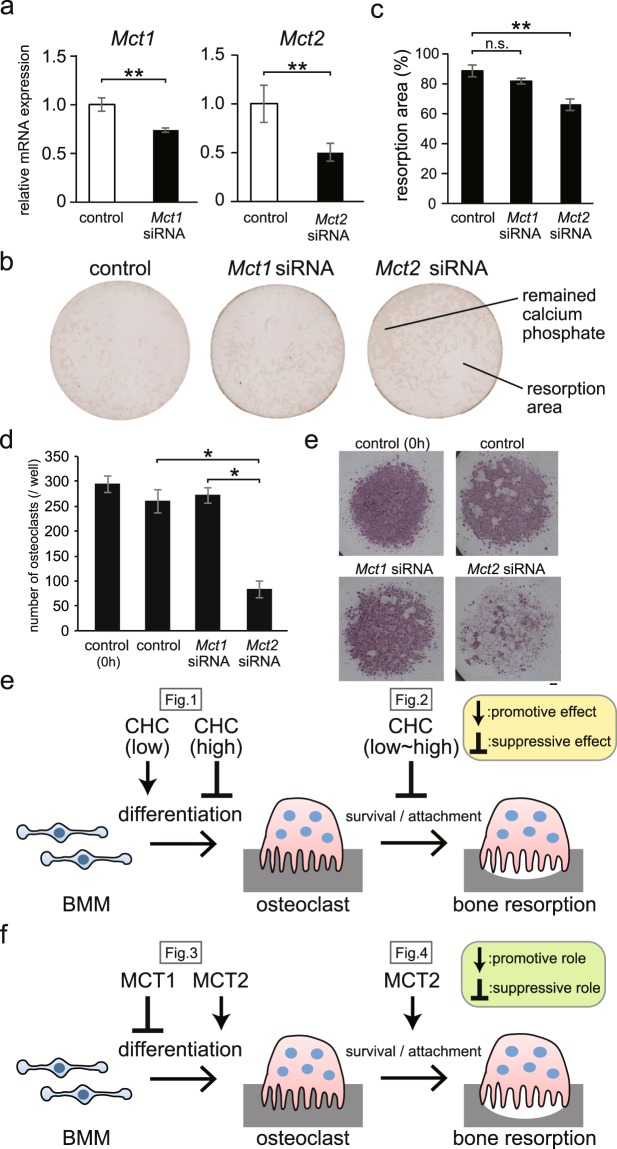
Effects of *Mct1* and *Mct2* gene silencing on the survival/attachment of mature osteoclasts. (**a–c**) BMMs were cultured in calcium phosphate-coated plates in the presence of M-CSF and RANKL for 72 hours, then *Mct1*, *Mct2*, or control siRNA was introduced, and culture continued for 24 hours. (**a**) At 24 hours after the introduction of siRNAs into osteoclasts, the expression of mRNA for *Mct1* and *Mct2* was quantitatively analyzed by real-time RT-PCR. Values were normalized by that of *Gapdh* and shown as relative to the control. (**b**) Representative photographs of plates at 24 hours after the introduction of siRNA. Lighter portions are areas of resorption. (**c**) Areas of resorption were quantified using the software package pre-installed in the optical microscope. (**d**,**e**) BMMs were cultured for 72 hours in the presence of M-CSF and RANKL to induce differentiated into osteoclasts in normal plastic plates. After counting and the initial number of osteoclasts (Start), they were introduced with *Mct1*, *Mct2*, or control siRNA, further cultured for 48 hours, and the number of osteoclasts was counted again (**d**). Osteoclasts were detected by TRAP activity staining (**e**). (**f**) Graphical depiction of a protocol to determine the effect of inhibition of MCTs on osteoclast differentiation and bone-resorbing activities. (**g**) Graphical depiction of a protocol to determine the effect of silencing of *Mct1* and *Mct2* genes on osteoclast differentiation and bone-resorbing activities. (**a,c,d**) Values are shown as the mean ± SD. * and ** indicate a significant difference at *p* < 0.05 and *p* < 0.01, respectively.

We also examined the effects of silencing of the genes for MCT1 and MCT2 in mature osteoclasts obtained after stimulation of BMMs for 72 hours by RANKL in order to clarify the role of these transporters in bone resorption. siRNA for *Mct1* as well as that for *Mct2* suppressed the expression of the corresponding genes in mature osteoclasts (Fig. 4a), whereas the expression of *Mct4* in osteoclasts was not suppressed by addition of its siRNA (data not shown). *Mct1* siRNA did not have any effect on the resorption of calcium phosphate by osteoclasts. On the other hand, that of *Mct2* significantly suppressed calcium phosphate resorption by mature osteoclasts (Fig. 4b,c). Furthermore, introduction of *Mct2* siRNA into mature osteoclasts that had been obtained by culturing BMM in the presence of RANKL reduced their number (Fig. 4d,e). These results indicate that MCT2 plays an important role in the osteoclastic bone resorption by supporting the survival of mature osteoclasts or their attachment to the bone surface.

## Discussion

The results obtained in this study are presented in a schematic form in Fig. 4f,g. CHC at a low concentration was shown to promote differentiation of BMMs into osteoclasts, whereas that at a high concentration suppressed it. Furthermore, CHC reduced the number of mature osteoclasts and hence suppressed the resorption of calcium phosphate in a concentration-dependent manner (Fig. 4f). Additionally, results obtained in experiments using specific siRNAs for MCT subtypes indicate that MCT1 is a negative regulator and MCT2 a positive regular of osteoclast differentiation of BMMs. The present study also found that MCT2, the expression of which is up-regulated during the process of osteoclast differentiation, is required for their survival or attachment to the surface of the plates or dentin discs (Fig. 4g).

CHC has been reported to inhibit of the 4 MCT subtypes (MCT1-4) that function to transport monocarboxylates^14^. Among those, the *Mct1*, *Mct2*, and *Mct4* genes are expressed in BMMs, though their expression varies following RANKL stimulation in a time-dependent manner, making it difficult to identify which is actually inhibited by CHC in BMMs. Nevertheless, our results showed silencing by the specific siRNA of each of the subtypes, thus indicating that the inhibitory effect of CHC towards BMMs is mainly via MCT1. On the other hand, in mature osteoclasts, the subtype inhibited by CHC is considered to be MCT2, as the mRNA of that was most highly expressed among the 3 subtypes at 72 hours after RANKL stimulation, while the introduction of *Mct2* siRNA to mature osteoclasts reduced the area of calcium phosphate resorption.

Previously, bone metastatic malignant tumor cells were shown to release lactic acid by an anaerobic metabolism function, which is then taken up by adjacent osteoclasts via MCT1, leading to lactic acid-induced bone resorption^15^. However, there may be differences regarding the expression of MCT subtypes between humans and mice, though that report noted that the bone resorption of osteoclasts was increased by lactic acid taken in via MCT1, while osteoclast differentiation was not affected. Our findings also indicate that exogenous lactic acid is necessary for survival of mature osteoclasts, because CHC decreased the number of mature osteoclasts and thereby suppressed the amount of bone being resorbed by them. MCT2 was shown to be required to take up monocarboxylic acid into cells^16^, which was also supported by our finding that *Mct2* expression increases with osteoclast differentiation. Furthermore, CHC caused a reduction in the number of mature osteoclasts (Fig. 2d–g). Since the subtype of MCTs mainly expressed in mature osteoclasts was MCT2, it is conceivable that CHC inhibited MCT2. Therefore, MCT2 is considered to be necessary for the survival of mature osteoclasts or their attachment to the surface of dentin discs and calcium phosphate-coated plates.

On the other hand, lactate dehydrogenase (LDH) was found to be activated in RANKL-stimulated BMMs, and stimulated the glycolytic and mitochondrial respiratory metabolisms^17^. In the present experiments, a decreased concentration of intracellular lactate was observed in BMMs following stimulation by RANKL (data not shown). Thus, an increase in endogenous lactate concentration based on a change in energy metabolism may promote osteoclast differentiation^17^.

A technical limitation of this study was our inability to determine the movement of lactic acid or other monocarboxylic acids across the cell membrane. If glycolytic progression could be observed^18^, as shown by NAD^+^/NADH ratio^19^, the resultant findings might indicate a correlation of energy metabolism with osteoclast differentiation and function^20^. Another technical limitation of this study was the inability to quantify the change in the bone-resorbing activity of a single osteoclast after the addition of CHC or the introduction of *Mct2* siRNA. Hence, we could not discriminate between the effects of these treatments on differentiation/survival and those on the bone-resorbing activity of osteoclasts.

The primary clinical and scientific implications of the present results are identification of the expressions and roles of MCT subtypes in osteoclasts. Additionally, we found that inhibition or silencing of transporters provides control of the differentiation and functsion of osteoclasts, indicating that transporters on the cell membrane of osteoclasts may serve as therapeutic targets for treatment of osteoporosis and other diseases related to bone metabolism.

## Methods

### Ethical approval statement

All of the present experimental protocols were approved by the ethical board for animal experiments of Showa University (approval number 17052), and conducted in accordance with Japanese Governmental Law (No. 10069) for the care and use of laboratory animals. The authors declare that all experiments were performed in accordance with relevant guidelines and regulations.

### Reagents

Recombinant human macrophage-colony stimulating factor (M-CSF) (Leukoprol^®^) and recombinant mouse receptor activator of nuclear factor κB (RANK) ligand (RANKL) were purchased from Kyowa Hakko Kogyo Co., Ltd. (Tokyo, Japan) and R&D Systems Inc. (Minneapolis, MN, USA), respectively. CHC (CAS number 28166-41-8), an inhibitor of MCT1, MCT2, MCT3, and MCT4, was purchased from Sigma-Aldrich (St. Louis, MI, USA). Collagenase, 1α,25-dyhydroxyvitamin vitamin D_3_ [1α,25(OH)_2_D_3_], prostaglandin E_2_ (PGE_2_), ScreenFect^TM^ siRNA, and Cellmatrix^®^ were purchased from FUJIFILM Wako Pure Chemical Co. (Osaka, Japan). Stealth^TM^ siRNAs for mouse *Mct1*, *Mct2*, and *Mct4*, and a negative control siRNA, as well as Alexa Fluor^TM^ 488 and 4’6-diamidino-3-phenylondole (DAPI) were obtained from Invitrogen (Carlsbad, CA, USA).

### BMMs

BMCs were isolated from the femurs and tibias of 5- to 8-week-old male ddY mice (Japan SLC, Shizuoka, Japan), and cultured for 72 hours in α-minimal essential medium (αMEM) (FUJIFILM Wako Pure Chemical Co.) supplemented with 10% fetal bovine serum (FBS) (Sigma-Aldrich), antibiotics, and M-CSF (4000 U/mL). Adherent cells were regarded as BMMs.

### Induction of osteoclast differentiation in BMM cultures

BMMs (1 × 10^4^ cells/well) were plated in the center of the bottom surface of 48-well normal plates. For some experiments, BMMs (2 × 10^4^ cells/well) were plated in 96-well normal plates or 96-well calcium phosphate-coated Osteo Assay Surface plates (Corning Inc., Lowell, MA, USA). They were cultured for 72 hours in αMEM supplemented with 10% FBS and antibiotics, in the presence of M-CSF (4000 U/mL) and RANKL (30 ng/mL), with or without various concentrations of CHC. To obtain mature osteoclasts, BMMs (1 × 10^7^ cells/well) were cultured for 72 hours at 37 °C in 10-cm temperature-sensitive RepCell^®^ plates (CellSeed Inc., Tokyo, Japan) in the presence of M-CSF (4000 U/mL) and RANKL (30 ng/mL). After incubation for 30 minutes at 4 °C, cells were collected as a suspension in the medium using centrifugation and used in the present experiments to examine the effects of CHC on mature osteoclasts.

### Induction of osteoclast differentiation in co-cultures of osteoblasts and BMCs

Mouse osteoblastic UAMS32 cells (1 × 10^6^ cells/well) and BMCs (2 × 10^7^ cells/well) were co-cultured in 10-cm dishes coated with collagen (Nitta Gelatin, Yao, Japan) for 8 days in αMEM plus 10% FBS and antibiotics, in the presence of 1α,25(OH)_2_D_3_ (10 nM) and PGE_2_ (1 μM). Cells were detached by incubation for 10 minutes at 37 °C with collagenase (0.4%) and dispase (0.2%) in αMEM, plus 10% FBS and antibiotics. Cells were collected by centrifugation and used for experiments to examine the bone resorption by osteoclasts.

### Introduction of siRNAs into BMMs and osteoclasts

Stealth^TM^ siRNAs for mouse *Mct1*, *Mct2*, and *Mct4*, as well as negative control siRNA were introduced into BMMs and osteoclasts using ScreenFect^TM^ siRNA by forward transfection. To induce osteoclast differentiation of BMMs, M-CSF and RANKL (50 ng/mL) were added to the medium at the same time as each siRNA was introduced.

### Bone resorption of osteoclasts

Osteoclasts obtained from BMM cultures in temperature-sensitive RepCell^®^ plates were re-plated in calcium phosphate-coated plates (Osteo Assay Surface, Corning Inc., Lowell, MA, USA), then cultured for 24 hours. Resorption pits were observed and quantified under an optical microscope (BZ-9000, Keyence, Osaka, Japan). Cells containing osteoclasts obtained in co-cultures of UAMS32 cells and BMCs were plated on dentin discs with a diameter of 6 mm, and cultured for 24 hours in 24-well plates. The dentin discs were sonicated in 5% ammonia in PBS to remove cells, then rinsed with water, air-dried, stained with 1% toluidine blue, and washed again with water. Resorption pits stained with toluidine blue were observed and quantified using an optical microscope (BZ-9000, Keyence).

### Real-time RT-PCR

Total RNA was extracted from cells using TRIzol^®^ reagent (Invitrogen), according to the manufacturer’s instructions. Reverse transcription reactions were performed using ReverTra ACE RT qPCR master Mix (TOYOBO Co. Ltd., Osaka, Japan). Quantitative real-time RT-PCR was performed using a TaqMan^TM^ Gene Expression Assay (Life Technologies, Carlsbad, CA, USA) and a StepOne Real-time PCR System (Applied Biosystems, Carlsbad, CA, USA). Amplification signals from the target genes were normalized against that of glyceraldehyde 3-phosphatase (*Gapdh*). The assay IDs for the genes of GAPDH, MCT1, MCT2, MCT3, MCT4, RANK, NFATc1, DCSTAMP, TRAP, Cathepsin K, and IRF8 were as follows: *Gapdh*, Mm99999915_g1; *Mct1*, Mm01306379_m1; *Mct2*, Mm00441442_m1; *Mct3*, Mm00445115_ m1; *Mct4*, Mm00446102_m1; *Rank*, Mmoo437135m1; *Nfatc1*, Mm00479445_m1; *Dcstamp*, Mm04209236_m1; *Trap*, Mm00475698_m1; *Ctsk*, Mm00484039_m1; and *Irf8*, Mm00492567_m1, respectively.

### Cell proliferation assay

BMMs were plated at a density of 1 × 10^4^ cells/well in 96-well plates, and cultured for various periods up to 72 hours in the presence of M-CSF (4000 U/mL) with or without CHC (0.1, 0.3, 1.0 mM). At the end of each culture period, cells were fixed with 4% paraformaldehyde in PBS, then washed with PBS and incubated for 20 minutes with DAPI solution (Invitrogen). After washing with PBS, cells were observed under a fluorescence microscope (BZ-9000, Keyence) and counted using the pre-installed software package.

### Tartrate-resistant acid phosphatase (TRAP) activity staining

Cells were fixed with 4% paraformaldehyde in PBS, then washed with PBS, and treated for 1 minute with a mixture of acetone and ethanol (1:1), washed again with PBS, and incubated for 30 minutes at 37 °C in TRAP buffer (pH 5.2) containing 0.2 mg/mL naphthol AS-MX phosphate (Sigma-Aldrich) and 0.6 mg/mL Fast Red Violet LB Salt (Sigma-Aldrich). After washing with pure water, they were observed under a microscope.

### Measurement of the number and area of osteoclasts

The number and area of TRAP activity-positive cells with a diameter of 200 μm or more were determined using a microscope (BZ-9000, Keyence) with analysis software (BZ-X Analyzer 1.3.1.1, Keyence).

### Determination of TRAP activity

Cells were homogenized in TRAP buffer with 0.1% Triton-X 100 (Simga-Aldrich) under sonication on ice. Cell lysates (20 μL) were added to 100 μL of TRAP buffer containing 3 mg/mL disodium *p*-nitrophenyl phosphate (FUJIFILM Wako Pure Chemical Co.). After incubation for 30 minutes at 37 °C, reactions were terminated by addition of 80 μL of 0.5 M NaOH, then absorbance of the reaction mixture at 405 nm was read using a microplate reader (SH-1000, Corona Electric, Ibaraki, Japan).

### Fluorescent staining of F-actin

Cells were fixed in 3.7% formaldehyde in PBS and treated with 0.5% Triton X-100 in PBS for 3 minutes. Next, cells were stained with 0.2 U/mL of FITC-phalloidin (Sigma-Aldrich) in 0.1% Triton X-100 for 1 hour. After additional staining with DAPI, they were observed under a fluorescence microscope (BZ-9000, Keyence).

### Cell viability assay

Live, dead, and apoptotic cells were quantified using a Tali^TM^ Apoptosis kit with Annexin V Alexa Fluor^TM^ 488 and propidium iodide (Thermo Fisher Scientific, Waltham, MA, USA), and a Tali^TM^ image-based Cytometer (Thermo Fisher Scientific).

### Statistical analysis

Values are expressed as the mean ± SD. Statistical analysis between 2 groups was performed using Mann-Whitney’s U test and between multiple groups using a Steel-Dwass test. *P*-values less than 0.05 were considered to indicate statistical significance.

## Supplementary information

Supplementary Information
