# Supplementary material for: Roles of monocarboxylate transporter subtypes in promotion and suppression of osteoclast differentiation and survival on bone
Source: Sci Rep. 2019 Oct 30;9:15608. doi: 10.1038/s41598-019-52128-2 (PMC6821745; doi:10.1038/s41598-019-52128-2)
Supplement: Supplementary file 1 — Supplementary Information [file 41598_2019_52128_MOESM1_ESM.pdf]

Supplementary Information

Roles of monocarboxylate transporter subtypes in promotion and suppression of osteoclast differentiation and function

Hiroko Imai[1, 2], Kentaro Yoshimura[1]*, Yoichi Miyamoto[1], Kiyohito Sasa[1], Marika Sugano[2], Masahiro Chatani[3], Masamichi Takami[3], Matsuo Yamamoto[2], Ryutaro Kamijo[1]

Departments of [1]Biochemistry, [2]Periodontology, and [3]Pharmacology, Showa University School of Dentistry, Tokyo, Japan

*Corresponding author: Department of Biochemistry, Showa University School of Dentistry, 1-5-8 Hatanodai, Shinagawa, Tokyo 142-8555, Japan
Tel: +81 3 3784 8163, Fax: +81 3 3784 5555, Email: kyoshimura@dent.showa-u.ac.jp

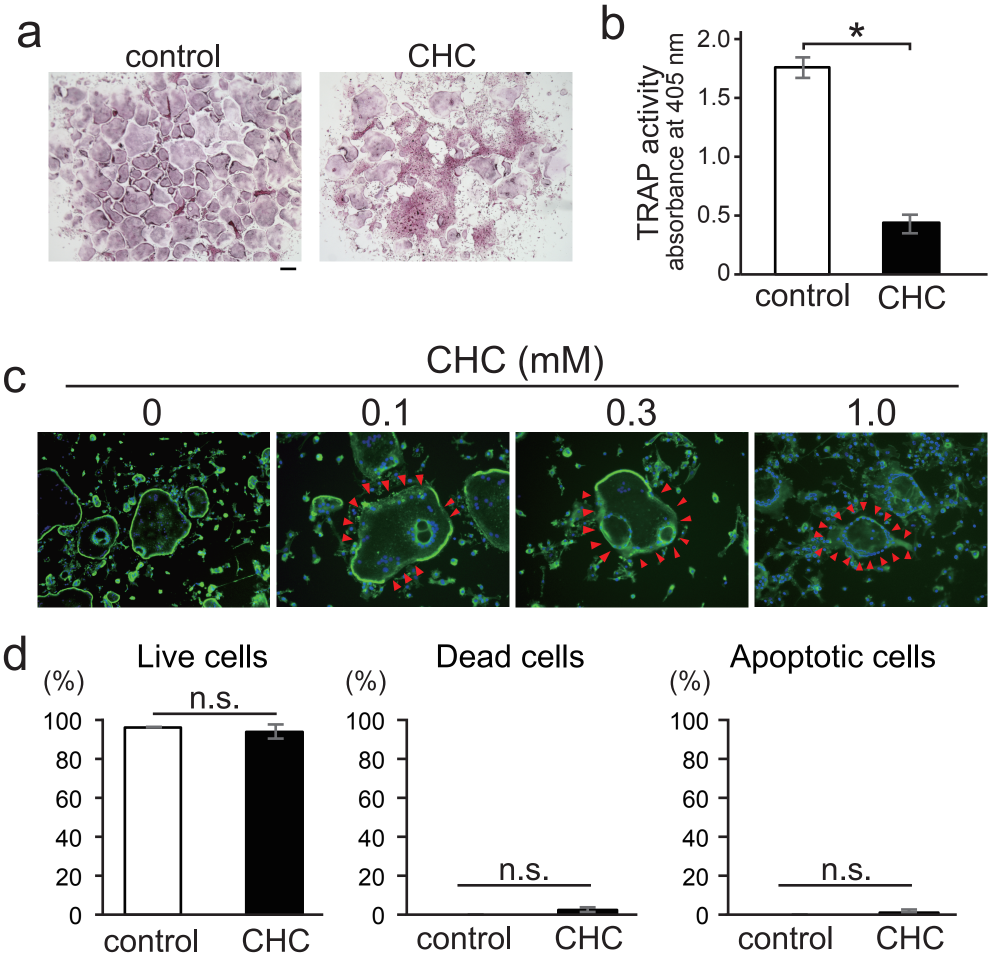

Supplementary Figure S1. Effect of CHC on mature osteoclasts. (a-d) BMMs were cultured for 72 hours in calcium phosphate-coated plates (a) or normal plastic plates (b-d) in the presence of M-CSF and RANKL, and presence or absence of CHC. (a,b) TRAP activity staining (a) as well as the determination of TRAP activity (b) were performed for cells cultured in the absence (control) and presence of CHC (0.3 mM). (c) Cells were stained with FITC-phalloidin and DAPI. Magnification, 10×. Loss of formation of actin rings is indicated by red arrowhead. (d) Proportions of live, dead, and apoptotic cells were determined after double staining with fluorinated annexin and propidium iodide. (a) Scale bars, 500 µm. (b, d) Values are shown as the mean ± SD. *Value significantly smaller than that obtained for the control without CHC ($p < 0.05$). n.s., not significant.
